# Endoscopic submucosal dissection for gastric ectopic pancreas: a single-center experience

**DOI:** 10.1186/s12957-019-1612-x

**Published:** 2019-04-16

**Authors:** Yangyang Zhou, Siran Zhou, Yang Shi, Shimeng Zheng, Bingrong Liu

**Affiliations:** Department of Gastroenterology, The First Affiliated Hospital of Zhengzhou University, Zhengzhou, 450000 China

**Keywords:** ESD, Ectopic pancreas, Gastric, Outcomes

## Abstract

**Background and objective:**

Endoscopic submucosal dissection (ESD) is a minimal invasive technology and could allow “en bloc” resection for superficial gastric tumors. The aim of this study is to evaluate the safety and feasibility of ESD for gastric ectopic pancreas (EP).

**Methods:**

A total of 93 patients diagnosed with ectopic pancreas who underwent ESD between January 2011 and June 2017 were enrolled. The demographic, clinical, and endoscopic data were collected and analyzed.

**Results:**

The average maximal diameter of lesions was 1.01 (range 0.4–3.0) cm with mean age of patients which was 39.75 (range 15–66) years. Overall, all of procedures en bloc was successful. The median operative time was 76.87 (range 30–160) min. A total of 12 patients experienced complications. In seven patients, bleeding occurred during the operation and was treated using hot biopsy forceps or metal clip. Five cases suffered from pneumoperitoneum which was managed well. The mean length of postoperative hospital stay was 5.7 (range 2–17) days. There was no relapse in any cases during the follow-up.

**Conclusion:**

ESD appears to be a safe and feasible approach for curative treatment in gastric ectopic pancreas. Larger studies are needed to identify the role and the outcomes of ESD in another center.

## Introduction

Ectopic pancreas is a congenital abnormal disease, and the incidence of EP ranges from 0.5 to 13% in the general population [1–3]. Ectopic pancreas could occur anywhere in the gastrointestinal tract, but the most common site is stomach [4]. Gastric ectopic pancreas is defined as the pancreatic tissue located in the stomach which does not continue to the pancreas and has independent innervation and vascular supply [5]. Upper abdomen pain or discomfort, acid reflux, nausea, and vomiting are the common symptoms of gastric ectopic pancreas while these symptoms are not specific. Thus, it is difficult to distinguish EP with gastric submucosal tumors from the clinical manifestation. The major conventional treatment is surgery or laparoscopic [6]. ESD is a minimally invasive approach for the treatment of digestive disease and has been widely applied [5]. ESD could allow “en bloc” and complete resection for superficial gastric tumors. And it could provide the pathological assessment preciously. However, previous studies reporting the safety and feasibility of ESD for EP were based on limited data [5, 7]. Therefore, the purpose of this study is to report 93 patients with EP treated with ESD in a single Chinese center. En bloc resection rate, operation results, analysis of complications, and recurrence at mid-long-term follow-up were assessed.

## Patients and methods

### Patients

From January 2011 to June 2017, more than 400 patients were diagnosed with ectopic pancreas by histopathological examination. Of these, 93 patients underwent ESD procedure for EP in the First Affiliated Hospital of Zhengzhou University. Endoscopic ultrasonography (EUS) was performed preoperatively to assess the size and origin of the lesions in the 67 cases. All patients who were enrolled in this study were informed about the benefits, disadvantages, and complications of the ESD techniques. And informed consent was signed by all participants. This study was performed in accordance with the Declaration of Helsinki and was approved by the institutional review board of our hospital.

### ESD procedure

Prior to endoscopic surgery, each patient had been evaluated by computed tomography and gastrointestinal endoscopy. But EUS was not performed routinely. Every patient was under general anesthesia during the operation. All patients were performed by expert endoscopists using a single-channel endoscope. Endoscopic surgery procedure was performed as follows.

1. Marking: First, make the marking dots, at a distance of 0.5 to 1.0 cm from the lesion; electrocoagulation was performed around the lesion with an argon knife.
2. Submucosal injection: In order to prevent complications such as perforation and bleeding, it is especially important to perform submucosal injection before operation to make the mucosa bulge. Injection was done on the outside of the lesion edge marker with a mixture of adrenaline and saline to the lesion.
3. Incision and dissection: The endoscopic hook knife was used to incise the mucosa along the lateral edge of the marked edge of the lesion, and then the transparent cap assists in stripping the lesion until the mucosal dissection is complete. Finally, the foreign forceps remove the tumors.
4. Check and fixed specimen: If there is bleeding during the operation, argon plasma coagulation (APC) and hot biopsy forceps can be used to stop the bleeding. It is found that the gastric defect can be closed with metallic clips. Lesion size was recorded.

### Histopathological assessment

Fix the resected specimen with the pin and place it in the formaldehyde to undergo pathological examination. Meanwhile, the size, lateral margins, basal margins, depth of invasion, and lymphovascular embolization were recorded and evaluated. If necessary, immunohistochemistry was applied for differential diagnosis. The definition of gastric HP is the presence of pancreatic tissue in the stomach. The histopathological diagnosis was affirmed by two expert pathologists.

### Follow-up

The day after the ESD operation, the complete blood cell count was measured and C-reactive protein was assessed. Endoscopy follow-up was done at 3, 6, and 12 months after the endoscopic procedure and yearly thereafter.

## Results

Clinical characteristics of all patients with EP were collected in Table 1. A total of 93 patients (47 men, 46 women) have an average age of 39.75 (range 15–66) years. The most common clinical symptoms were abdominal pain (40.86%), followed by abdominal distension (19.35%), abdominal discomfort (16.13%), asymptomatic EP (16.13%), acid reflux (9.68%), and nausea (4.3%).

**Table 1:** Baseline characteristics of 93 patients with ectopic pancreas

Patients	*N* (%)
Age, median (range)	39.75 (15–66)
Sex	
Male	47 (47/93)
Female	46 (46/93)
Clinical symptoms	
Abdomen pain	38 (38/93)
Abdomen distension	18 (18/93)
Abdominal discomfort	15 (15/93)
Acid reflux	9 (9/93)
Nausea	4 (4/93)
Asymptomatic	15 (15/93)
Tumor subsites	
Gastric antrum	74 (74/93)
Gastric body	18 (18/93)
Gastric angle	1 (1/93)
Size of the tumors, median (range)	1.01 (0.4–3.0)
≤ 1 cm	58 (58/93)
> 1, ≤ 2 cm	31 (31/93)
> 2 cm	4 (4/93)

Overall, all procedures were done according to ESD criteria. Seventy-four lesions were located in gastric antrum (79.57%), 18 in the gastric body (19.35%), and 1 in the gastric angle (1.08%). The median size of lesion is 1.01 (0.4–3.0) cm. In details, more than half of lesions were ≤ 1 cm (62.37%), 31 lesions were between 1 to 2 cm (33.33%), and 4 lesions were larger than 2 cm (4.3%).

Table 2 summarizes the outcomes of EP resected by endoscopic submucosal dissection. One of the characteristics of EP in the endoscopy is umbilication. In our center, 51 lesions present umbilication during the endoscopy procedure and the rest without umbilication. EUS is an important examination which could determine the origin of tumors. And 67 cases received EUS before operation. Most of the lesions originated from the submucosa (53.76%), followed by the mucosa (11.83%) and the muscularis propria (6.45%). In all cases, en bloc resection was achieved successfully. The mean operation time was 76.87 (range 30–160) min. The histopathological results of the lesions were confirmed EP by two expert pathologists.

**Table 2:** Outcomes of ectopic pancreas resected by endoscopic submucosal dissection (ESD)

Patients	*N* (%)
Umbilication	
With umbilication	51 (51/93)
Without umbilication	42 (42/93)
Preoperative EUS to determine the origin	
The mucosa	11 (11/93)
The submucous	50 (50/93)
The muscularis propria	6 (6/93)
Not examined	26 (26/93)
En bloc rate	93 (93/93)
Time of operation, median (range)	76.87 (30-160)
Complication	
Pneumoperitoneum	5 (5/93)
Bleeding	7 (7/93)

Overall, 12 patients (12.9%) suffered complication. Five cases (5.38%) experienced perforation during the operation and were cured in time. In 7 patients (7.53%), bleeding occurred and was controlled by applying hot biopsy forceps or metal clips. Each of the patients discharged successfully and had no recurrence during the median 42.06 (16–82) months follow-up period.

## Discussion

EP is a relative rare disease which could occur in the digestive system [8]. The most common location of EP is the stomach [4]. The results of the present study show that ESD is an alternative approach for treating gastric EP. Therefore, the aim of this study is to assess the safety and feasibility of ESD for gastric EP in a mid-long-term follow-up period. A previous study reported that it is more common in males than females compared to our study [9]. Gastric EP is found in the antrum in 85% of patients, and it could be located at either the anterior or the posterior wall. Meanwhile, it has showed that the origin of EP is mainly submucosa. In our survey, 79.57% of EP were located in the antrum and 53.76% originated from the submucosa.

Clinical symptoms of EP are not typical, and the abdominal pain is commonly found [1, 5, 10]. However, the factor causing abdominal pain remains unknown. Gastric EP is usually found occasionally during the gastroscopy, but clinical symptoms may occur depending on its size and pathological changes [4]. Rare complications caused by gastric EP have been reported, such as gastric outlet obstruction, bleeding, and ulceration [11, 12]. In our cases, 40.86% of patients present abdominal pain and 16.13% with abdominal discomfort. This corresponds to results in other studies [4, 7, 10].

In terms of gastric EP, most of them were relatively small and the clinical manifestations were not obvious [13]. Gastric EP has three common characteristics to help identify it. The first characteristic is the site in the gastric antrum [4, 14, 15]. In our materials, 74 of out 93 lesions locate at gastric antrum. The second feature is central umbilication [16]. This is a symbol of gastric EP and is the opening of excretory tube which could secrete hormone. Nevertheless, the lesions larger than 15 mm often could show central umbilication, and the lesion might be misinterpreted for the other tumor or another malignancy [1]. Therefore, it is challenging in the diagnosis of relatively small gastric EP. In our cases, more than half of tumors were smaller than 1 cm and 51 of 93 patients show the central umbilication during the gastroscopy examination. This is consistent with the description of other scholars [16–18]. The third attribute is the lesions had the complete mucous membrane. All cases in our center were an intact mucous membrane which is similar to the reported literature [1].

Currently, there are some limitations in diagnosing gastric EP. The imaging examination such as computed tomography and barium meal seems not very helpful in the diagnosis of gastric EP [19]. Pathological diagnosis is the gold standard. However, histologic diagnosis based on forceps biopsy is not adequate for determining endoscopic treatment of gastric EP. Biopsy may lead to wrong results sometimes [4, 6]. The histopathological finding of biopsy before operation showed the normal gastric mucosa, but demonstrated gastric EP postoperation. The reason of this may be that the biopsy is often shallow and most of the EP are located in the submucosa or muscularis propria. Thus, the accurate diagnosis of gastric EP is difficult as there was no specific examination applied before resecting the lesions. For precise pathological diagnosis, scholars have made a lot of attempts and explored many novel approaches to get the endoscopic techniques obtaining deeper specimens, such as EUS-guided biopsy [20–22]. Endoscopic resection is also an option to accurate diagnosis and treatment for gastric EP.

In recent years, with the wide application of endoscopic ultrasonography (EUS), it has showed unique advantages in the diagnosis of gastric EP [13]. We could perform a needle biopsy under EUS guidance, which could obtain more accurate specimens. Then, it is advantageous to accurately determine the nature, origin, and size of the tumor before endoscopic surgery and determine the more suitable approach for the patient which could reduce the patient’s trauma and bring more minimally invasive benefits to the patient [21, 23]. In addition, it is generally believed that EP is more common with mixed echoes. Some scholars reported that the overall echo is low and most lesion boundaries under EUS are unclear and irregularly lobulated [21, 24]. There were 57 patients with gastric EP performed EUS, and the conclusion was met with the article published. Therefore, EUS could be applied routinely in the examination of gastric mass.

The guidelines of gastric EP are still under controversial [15]. There are three main treatment methods at present: surgery resection, endoscopic resection, and endoscopy surveillance. If the pathological diagnosis of gastric EP has been made before operation of lesions located in the muscularis propria or serosal layers or obvious symptoms, the lesions could be resected. However, it may reduce the quality of life and increase the risk of long-term complications [25]. Consequently, in terms of asymptomatic gastric EP, some scholars recommended regular follow-up [26]. Nevertheless, regular follow-up will also cause some problems for the reasons as follows. First, once the patient knew about the lesion, the psychological burden will increase dramatically and urge the doctor to resect the lesion. Second, due to poor economic and limited medical conditions, several patients cannot afford to go to the big hospital and the cost of relevant examinations. Third, multiple endoscopy examination also increased the psychological burden. At our endoscopy center, 15 patients with no obvious symptoms underwent ESD surgery for above reasons. During an average of 42 months of follow-up, no endoscopic surgery-related complications and recurrence occurred. And at the time of telephone follow-up, the patients all showed that the anxiety was significantly reduced. In the previous study, Zhou [7] and Ryu [1] have reported that endoscopic management for asymptotic gastric EP is feasible and safe with good prognosis. Therefore, we thought that ESD techniques could be applied for asymptotic gastric EP if they have demand.

To date, laparoscopic surgery is an effective and safe treatment method and has been the major therapeutic option for gastric EP [25]. However, laparoscopic surgery has its own complications and limitations. Especially, it will be difficult to perform if the lesions were located near or at the pylorus and could result in fluid reflux or stenosis. Thus, ESD as a minimally invasive technique is suitable for treating gastric EP.

In our endoscopy center, on the basis of large successful endoscopic surgery experience, we have undergone ESD for gastric EP. In the total of 93 patients, all lesions were resected successfully and en bloc was achieved in each patient. The average duration of operation is 76.87 min which is longer than other studies. The possible reason is that in the early stage of ESD, endoscopist spent much time on dissection. As in that of others [4, 7], all of symptomatic patients experienced symptom relief after endoscopic surgery.

Ectopic pancreatic tissue without capsule is mainly originated from the submucosa of the gastrointestinal tract. And it could also invade into the muscularis or be closely associated with the muscularis. Therefore, complete resection is the key to its treatment and prevention of recurrence. Lee [27] and Zhang [28] had demonstrated that ESD could be used for the resection of gastric subepithelial tumors originating from muscularis propria layer. Among our cases, six patients with gastric EP originating from muscularis propria underwent ESD. They were resected successfully, and no recurrence occurred during the follow-up period. Moreover, one patient suffered perforation and was managed well with metal clip and recovery smoothly. In our experience, we should observe carefully before dissecting and sutured the leak with clips or stitched equipment calmly. Thus, ESD could offer a reliable option, allowing definitive management of SMTs with relatively low risk of complications.

On average 6 days, all patients discharged smoothly and had no recurrence during the mid-long-term follow-up. Based on our endoscopy center experience and review of the literature, we suggest that symptomatic gastric EP, asymptomatic EP larger than 1 cm, lesions located in deep layer, or suspected malignant could be treated using ESD procedure.

In addition, the rate of complications during the ESD procedure is relatively low. The common complication of ESD is perforation and bleeding [4]. In our study, 12 patients suffered slight complication but recovered smoothly. Perforation occurred in five patients, and seven patients suffered from bleeding. The pneumoperitoneum occurred when the lesions located at deep layer during the ESD operation. We will use clips only or combine nylon loop to close the wound. And the puncture needle was applied to relief the symptoms of abdominal distention. Of the 5 perforated patients, 3 were clipped with metal clips and 2 with metal clips and nylon sutures. Intravenous nutrition, antibiotics, and gastrointestinal decompression were applied after endoscopic operation. A review of the gastroscope after 3 months showed that the wound healed well. Based on our experience, we recommend that when dissecting the deep lesions, the edge of the lesion could be pulled under the traction of the snare to fully expose the lesion, thereby having a clear view and reducing the occurrence of perforations. Bleeding is more common than perforation which could be controlled by hot biopsy forceps or metal clips. There were 6 cases of intraoperative bleeding in this survey. For patients with intraoperative bleeding, we used saline flushing combined with hot biopsy forceps to adequately control bleeding. And there was 1 case of delayed bleeding after operation in this group. The lesion was located on the anterior wall of the stomach, with a maximum diameter of 25 mm. Due to the relatively large lesion and since it was performed at the early period of this study, it would be more difficult to perform than the others. After that, we practice the techniques and find that we could avoid dissecting the obvious small arterial blood vessels when operating. If necessary, metal clips could be used to clamp the blood vessels. Last but not the least, the wounds were carefully screened after surgery and washed thoroughly with saline to confirm at the end of surgery without obvious bleeding points. The EP often has 2 to 3 thick nourishing blood vessels which is the blood supply to the EP.

The above vessels should be treated carefully and predictably during endoscopic surgery which could reduce the rates of bleeding during or after surgery.

The rate of complication is 12.9% which is higher than other studies [29]. We found that these patients with complications were mainly at the early stages of this study. After gaining enough endoscopic surgery experiences, the duration of surgery and the incidence of complications were significantly reduced.

In this study, we enrolled 93 cases of patients with 93 lesions, all of whom underwent management of ESD. We found that ESD was not only feasible and safe for gastric EP, but also has advantages of being minimally invasive and beautiful. All lesions were resected successfully without additional surgery; the average operational time was 76.87 min, which was comparable to other studies [5, 7, 15]. This study provide an optional choice to treat gastric EP which is met with Zhong [7] who have reported that endoscopic treatment is suitable for gastric EP and showed the safety and feasibility of endoscopic techniques for gastric EP in 60 cases. Meanwhile, Sisman [30] and Gong [31] have revealed ESD could be applied for duodenum EP or with pancreatitis and pseudocyst formation. Therefore, we believe that as long as we are skilled in hemostasis and suturing techniques, ESD could be applied to the gastric EP.

Our study presents some limitations. First, it was a retrospective study and we had not performed propensity score matching. In addition, we did not account for some basic factors, such as cardiovascular disease, diabetes, and status of health conditions. Second, even though we enrolled a relatively larger number of cases, this is just a single-center experience and lack of power could be a reason for the lack of significant difference. Nevertheless, an advantage of our study was that it enrolled a relatively large number of patients to prove the safety and feasibility of ESD for gastric EP. Although several studies have reported the short-term outcomes, this is a rare study to assess the mid-long-term survival in patients who underwent ESD.

In conclusion, the results of the present study suggest that ESD for gastric EP is a safe and feasible technique when performed by experienced endoscopists. Meanwhile, the random controlled trials further needed to confirm the results.

## Abbreviations

EP: Ectopic pancreas
ESD: Endoscopic submucosal dissection
EUS: Endoscopic ultrasonography
